# Study on Material Flow Behavior in Three-Dimensional Directions During Friction Stir Welding and the Establishment of a Qualitative Model

**DOI:** 10.3390/ma19071341

**Published:** 2026-03-27

**Authors:** Cheng-Gang Wei, Sheng Lu, Jun Chen, Jun Zhang, Jin-Ling Zhu, Alexander V. Gridasov, Vladimir N. Statsenko, Anton V. Pogodaev

**Affiliations:** 1 School of Materials Science and Engineering, Jiangsu University of Science and Technology, Zhenjiang 212003, China; 181060001@stu.just.edu.cn (C.-G.W.); 192060016@stu.just.edu.cn (J.C.); 2 National Mold Inspection and Testing Center, Kunshan 215300, China; zjbaggio@163.com (J.Z.); zhujinling981113@gmail.com (J.-L.Z.); 3 School of Engineering, Far Eastern Federal University, Vladivostok 690950, Russia; gridasova.ea@dvfu.ru (A.V.G.); vladsta@mail.ru (V.N.S.); pogodaev.av@dvfu.ru (A.V.P.)

**Keywords:** friction stir welding, material flow, marker material, force-flow coupling model, flow with circulation

## Abstract

**Highlights:**

- The complex flow behavior of metals around the stirring tool during the welding process has become a core physical process affecting welding quality and process stability.
- Three methods of marker material configuration were adopted to investigate the three-dimensional material flow behavior during friction stir welding, and a “force-flow coupled simple circulatory flow” model was proposed.
- The experimental results indicate that three typical characteristic zones exist along the vertical direction, which are the shoulder-affected zone, the pin-affected zone, and the swirl zone from top to bottom.
- The “force-flow coupled simple circulatory flow” model defines three flow modes corresponding to the different characteristic zones within the weld.
- This study provides an interpretable qualitative basis for the three-dimensional material flow in friction stir welding.

**What are the main findings?**

- Three typical characteristic zones exist along the vertical direction, which are the shoulder-affected zone, the pin-affected zone, and the swirl zone from top to bottom.
- The material in the shoulder-affected zone is dominated by laminar flow; the pin-affected zone exhibits complex mixed-flow characteristics; while the swirl zone shows an obvious rotational flow pattern.
- A “force-flow coupled simple circulatory flow” model was proposed, which defines three flow modes corresponding to the different characteristic zones within the weld.

**What are the implications of the main findings?**

- It reveals that there exist substantial differences in the three-dimensional material flow behavior.
- The formation of welding defects is the result of the superposition of abnormal flows in different material flow zones.
- The model presents the qualitative relationship between welding process parameters and material flow behavior, and subsequent modeling work will help control welding quality by directly regulating the process parameters.

**Abstract:**

The complex flow behavior of the metal around the stirring tool during welding directly determines the microstructural evolution, defect formation, and mechanical properties of the welded joint, and thus becomes the core physical process affecting welding quality and process stability. In this study, to characterize the three-dimensional material flow behavior of AZ31 magnesium (Mg) alloy during friction stir welding (FSW), conventional metallographic sectioning was adopted as the primary observation method, and copper foil was used as the marker material. The flow trajectories of the materials after welding were investigated via three configurations of the marker material. The results indicate that three typical characteristic zones exist along the vertical direction, which are the shoulder-affected zone (SAZ), the pin-affected zone (PAZ), and the swirl zone from top to bottom. Specifically, the material in the SAZ is dominated by laminar flow; the PAZ exhibits complex mixed-flow characteristics; while the swirl zone shows an obvious rotational flow pattern. Based on the principles of material mechanics and fluid mechanics, a force-flow coupled “simple flow model around a rotating cylinder” was proposed, which defines three flow modes corresponding to the different characteristic zones within the weld.

## 1. Introduction

As a revolutionary solid-state joining technology, friction stir welding (FSW) has demonstrated enormous application potential in aerospace, rail transit, shipbuilding and other fields since its invention by the Welding Institute (TWI) of the United Kingdom in the early 1990s [1,2,3]. By plunging a high-speed rotating stirring tool into the workpiece and moving it along the weld, this technology achieves material joining through frictional heat and plastic deformation, thus avoiding defects such as porosity and cracks that are common in conventional fusion welding [4,5,6].

However, in contrast to any other welding processes, FSW requires careful selection of process parameters to ensure the consistency and repeatability of defect-free welds as well as their microstructures and mechanical properties. Generally speaking, the defects generated during the FSW process are mainly classified into flow defects and geometric defects [7,8], among which flow-related defects are the most common, including flash formation, surface abrasion, tunneling, wormholes, and lack of consolidation [9,10,11]. Flow defects mainly originate from the decreased material flowability caused by insufficient heat input (e.g., excessively low rotation/welding speed ratio), which prevents the transient cavity behind the pin from being timely filled by plasticized material [12]. In addition, the residual stress field generated during welding may further aggravate the evolution of the above defects by affecting the yield behavior and flow path of local material [13]. Therefore, the complex flow behavior of the metal around the stirring tool during welding directly determines the microstructural evolution, defect formation, and mechanical properties of the welded joint, and thus becomes the core physical process affecting welding quality and process stability [14,15].

The formation of the welding flow field involves plastic material flow under high-temperature and high-strain-rate conditions, and it is a nonlinear process characterized by strong coupling of multiple physical fields. The geometric characteristics of the stirring tool (e.g., shoulder diameter, pin profile), process parameters (rotational speed, welding speed, plunge depth), and material properties jointly act on the flow field, endowing it with highly transient and three-dimensional features [3,16]. For a long time, the invisibility of material flow has rendered its direct observation and quantitative characterization a technical bottleneck and cutting-edge challenge in this field. To address this issue, researchers have conducted extensive studies, including welding dissimilar materials [17,18,19], introducing marker materials into the weld [20,21], and adopting techniques such as X-ray imaging or computed tomography (CT) scanning to observe material flow within the weld [22,23,24], which have yielded certain achievements.

Colligan [25] tracked material flow in 6061 and 7075 aluminum alloys by embedding small steel balls in grooves cut parallel to the welding direction on the workpiece. The results showed that the material impinging on the pin at the advancing side (AS) of the weld was swept around the rotating pin and often deposited at the retreating side (RS) behind the pin. Generally (depending on its depth), the material impinging on the pin at the RS was also deposited at the RS behind the weld. Kumar et al. [26] proposed a special experiment in which the interaction between the FSW tool and the base material was continuously increased. The results indicated that there were two distinct material flow modes during FSW process, namely the pin-driven flow and the shoulder-driven flow. Recently, Liu et al. [27] conducted two FSW experiments using a rotating tool, one from Al 6061 to Al 1050 and the other from Al 1050 to Al 6061. They adopted the tool emergency stop technique to preserve the transient state of material flow around the tool. The results demonstrated that a rotating flow zone formed around the tool during welding. Most of the tracer marks were observed under the shoulder and around the pin, with only a small portion distributed at the bottom of the pin. Huang et al. [28] placed T3 copper foils at the top, middle, and bottom layers of the aluminum plates to investigate the vertical material flow behavior. The results showed that during FSW, the downward and upward material flows converged at the AS, altering the morphology of the nugget zone (NZ); the NZ exhibited a four-layer stacked structure, corresponding to the four vertical threads, which might be attributed to material deposition behind the rotating pin. In addition, Agiwal et al. [24] employed high-speed X-ray imaging technology to capture the in situ process dynamics of FSW during the welding of 6061-T6 aluminum alloy plates. The results revealed that the material filled the cavity in the tool wake and formed the weld every three tool rotations. At the current stage, numerous researchers have adopted computer numerical simulation techniques to investigate material flow and heat generation during the FSW process, and a variety of models have been proposed [29,30,31,32]. Different experimental results have revealed several universal characteristics of material flow in FSW. The material is forced to flow around the RS of the pin, resulting in an asymmetric flow field [3]. Up to date, although certain progress has been made in flow field research, and some experimental observations and simulation results have been widely recognized, previous studies have mostly focused on the material flow behavior near the nugget zone or along the vertical direction of the weld. For example, the single-layer marker method has been used to obtain two-dimensional cross-sectional flow trajectories, while dissimilar material welding enables the inference of material flow based on composition distribution. However, systematic research on the three-dimensional flow characteristics in the thickness direction—especially the migration paths of materials at different depths, mixing mechanisms, and their correlations with process parameters—is still lacking. This limits the in-depth understanding of the microstructure formation mechanism in the joint. The systematic investigation of the three-dimensional material flow behavior is the objective of this study.

The purpose of this study is to investigate the three-dimensional material flow behavior during FSW by embedding marker materials at appropriate positions, and to roughly delineate the three-dimensional material flow trajectories. Through the stress analysis of the material near the welding tool interface, a simplified model is proposed to qualitatively explain the three-dimensional material flow behavior on the basis of existing material flow theories.

## 2. Materials and Methods

All workpieces processed by FSW in the present study were commercial rolled AZ31 Mg alloy (Xintai Instrument Co., Ltd., Dongguan, China) with a nominal chemical composition of Mg-3.0Al-1.0Zn (wt. pct). In addition, varying quantities of 0.1 mm-thick T2 copper foils (Biling Co., Ltd., Taizhou, China) were inserted into the workpieces in different configurations as marker materials. These two materials can be easily distinguished under an optical microscope, facilitating the observation of the flow patterns of the marker materials.

**Figure 1:**
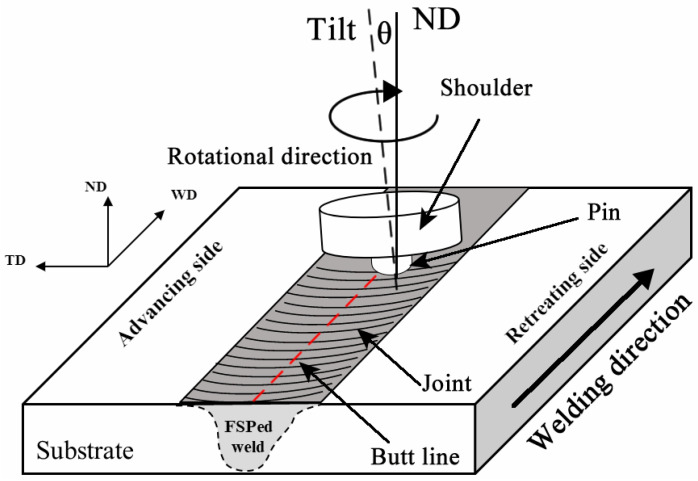
A schematic of the FSW process indicates the process’s main characteristic features.

FSW was performed using a friction stir welding machine (FSW-3LM-002, FSW Technology Co. Ltd., Beijing, China) in this study. The welding tool was fabricated from H13 tool steel (FSW Technology Co. Ltd., Beijing, China), consisting of a concave shoulder and a tapered pin with a length of 3.85 mm and an M6 left-hand threaded profile. The diameters of the shoulder and the pin were 16 mm and 4 mm, respectively. During the welding process, the plunge depth of the pin was controlled at approximately 3.9 mm, indicating that the shoulder of the welding tool was plunged 0.05 mm below the surface of the workpiece plate. In addition, the tool tilt angle relative to the normal direction (ND) was set at 2.5°. Regarding the process parameters, the tool rotational speed was 1500 revolutions per minute (rpm) and the traverse speed was 50 mm/min. These parameters were selected based on the results of our previous work [33]. Figure 1 is a schematic diagram of the FSW process. It should be noted that the critical directions of the FSW geometry are defined as follows: welding direction (WD) in the workpiece, rolling direction (RD), and normal direction (ND), as presented in Figure 1.

**Figure 2:**
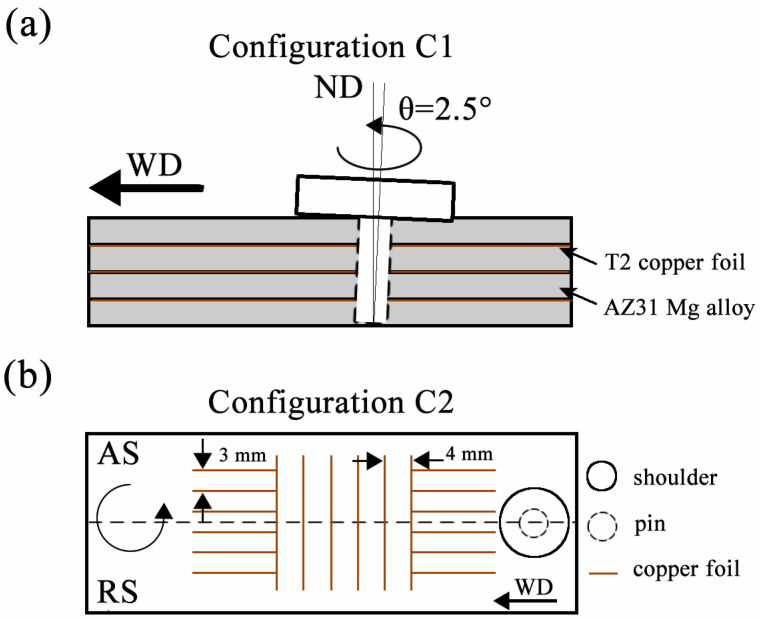
Schematic view of marker materials placement in two configurations: (**a**) Configuration C1 and (**b**) Configuration C2.

To comprehensively characterize the final three-dimensional material flow induced by the stirring tool (including the shoulder and pin) in the post-weld zone of FSW, two configurations were adopted in the marker material visualization experiments. In Configuration 1 (denoted as C1), four rolled AZ31 Mg alloy sheets (200 L × 60 W × 1 T mm) were laminated together, with a T2 copper foil (200 L × 60 W × 0.1 T mm) inserted between each adjacent pair. The specimen was clamped in a fixture, and then FSW was performed along the longitudinal direction (welding direction, denoted as WD) perpendicular to the rolling direction (RD) at the midpoint of the entire specimen, forming a 50 mm-long welded zone. Figure 2a illustrates the design method of C1. This configuration effectively enables the acquisition of material flow characteristics within the entire welded zone after the longitudinal traversal of the stirring tool. In Configuration 2 (denoted as C2), T2 copper foils with a thickness of 0.1 mm and varying lengths were embedded symmetrically along the midline of the WD in both the transverse and longitudinal directions into the prepared AZ31 Mg alloy plate (200 L × 60 W × 4 T mm), as shown in Figure 2b. The stirring tool was first plunged into the workpiece containing the longitudinal copper foil, and after 15 mm of processing, it was moved to the first horizontal copper foil. Subsequently, the stirring tool advanced a distance of 20 mm, passing continuously through six horizontal copper foils and contacting the longitudinal copper foil again until the end of the process, forming a complete 50 mm-long welded zone. The fundamental purpose of Configuration C2 is to investigate the material flow characteristics of plastic deformation in the horizontal direction of AZ31 Mg alloy around the stirring tool and within the fully welded zone at different welding depths during the FSW process. Figure 2a,b depict and summarize the configurations of the two experimental designs as well as the positions of the marker materials in each specimen.

During the experiment, the three-dimensional (3D) reaction forces, including the longitudinal force (Fx), lateral force (Fy) and axial force (Fz), were monitored in real time using self-developed equipment, to evaluate the effect of artificially added marker material on material flow behavior in the welding zone. The positive direction of the X-axis was defined as the tool feed direction, while the positive direction of the Y-axis was set from the AS to the RS. The positive direction of the Z-axis was set as the vertically downward direction.

After FSW, the processed workpieces were cut into specific specimens, which were observed and investigated using an optical microscope (OM). For C1, a typical transverse cross-section adjacent to the pinhole at the end of the welded joint was selected and cut from the workpiece into small test pieces with dimensions of 30 L × 10 W × 4 T mm. In addition, the welded joint was cut into two parts along the weld centerline, and a longitudinal cross-section specimen (30 L × 10 W × 4 T mm) adjacent to the AS was taken along the longitudinal direction at the middle of the weld for longitudinal flow observation. For C2, electrical discharge machining (EDM) was performed in the horizontal direction (WD-RD plane) at distances of d = 1.0 mm, 2.0 mm, and 3.0 mm from the top surface of the weld, respectively. After machining, metallographic grinding was conducted using silicon carbide (SiC) papers of different grit sizes, followed by polishing with diamond paste. The polished specimens were etched with a mixed solution consisting of 5.0 mL nitric acid, 2.0 g oxalic acid, and 100 mL distilled water, and then rinsed with ethanol. Material flow in three directions was observed using an ultra-depth three-dimensional microscope (VHX-900, Keyence, Osaka, Japan) and a Zeiss metallographic optical microscope (Zeiss Axioskop 2 MAT, Oberkochen, Germany). To effectively identify the distribution of Mg and Cu elements in the C2 specimens, scanning electron microscopy coupled with energy-dispersive X-ray spectroscopy (SEM-EDS, Shimadzu, JSM-6480, Kyoto, Japan) was employed for sample analysis.

## 3. Results and Discussion

### 3.1. The Results of 3D Reaction Forces

In general, material flow during FSW is an extremely complex and invisible three-dimensional behavior. It is governed by tool geometry, welding parameters, workpiece material properties, the contact condition at the tool/matrix interface, and even surrounding environmental conditions such as ambient temperature. It can be concluded that even a slight variation in any of the above factors can significantly modify the material flow pattern around the pin. Therefore, it is essential to evaluate the influence of marker materials embedded within the weld zone on the key welding/processing parameters that represent the FSW process.

As is well-known, FSW is an extremely complex “thermo-mechanical coupling” process. Obviously, monitoring the variation trend of forces during the welding process can directly and clearly reflect the influence of changes in certain experimental factors on the key welding parameters [34]. Therefore, by comparing the differences in the monitored forces, the influence of markers on material flow during the FSW process can be indirectly evaluated when the stirring tool moves from the marker-free zone to the marker-containing zone.

**Figure 3:**
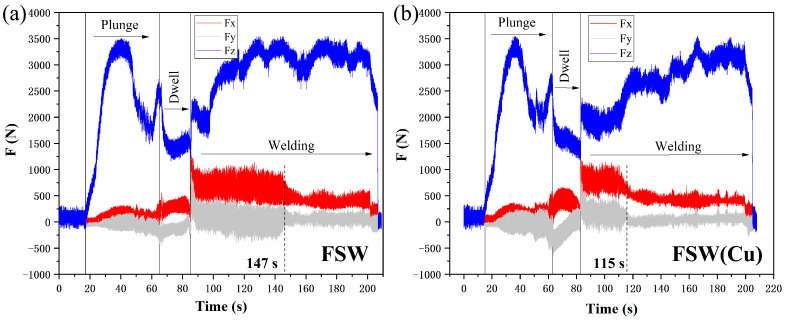
The variation trends of the three-dimensional reaction forces on the workpiece during the entire welding process. (**a**) FSW condition; (**b**) FSW (Cu) condition.

Figure 3 shows the variation trends of the three-dimensional reaction forces on the workpiece during the entire welding process. For the conventional FSW process (without marker material), according to the interaction with the workpiece, the variation in the three-dimensional forces can be divided into three stages (as shown in Figure 3a), namely plunging (Stage 1), dwelling (Stage 2), and welding (Stage 3). It can be observed that compared with the axial force (Fz), the drag force (Fx), and lateral force (Fy) exhibit no significant changes. In particular, the lateral force fluctuates slightly around zero throughout Stage 3, indicating that the process enters a stable welding stage. When the marker material (T2 copper foil) is inserted into the workpiece (Figure 2a), no obvious changes in the three-dimensional reaction forces are detected during the welding stage (Stage 3), as shown in Figure 3b. Interestingly, conventional FSW takes approximately 32 s longer than FSW (Cu) to reach the stable welding stage, suggesting that FSW with copper foil achieves a stable state earlier. Excluding measurement errors, this indicates that the material flow pattern revealed by the marker material is similar to the conventional flow pattern. Furthermore, similar results have been reported in Ref. [34]. Therefore, the influence of a suitable marker material on the plastic flow of the base metal can generally be neglected in the present work.

### 3.2. Vertical and Longitudinal Flow—Configuration C1

#### 3.2.1. Vertical Flow

**Figure 4:**
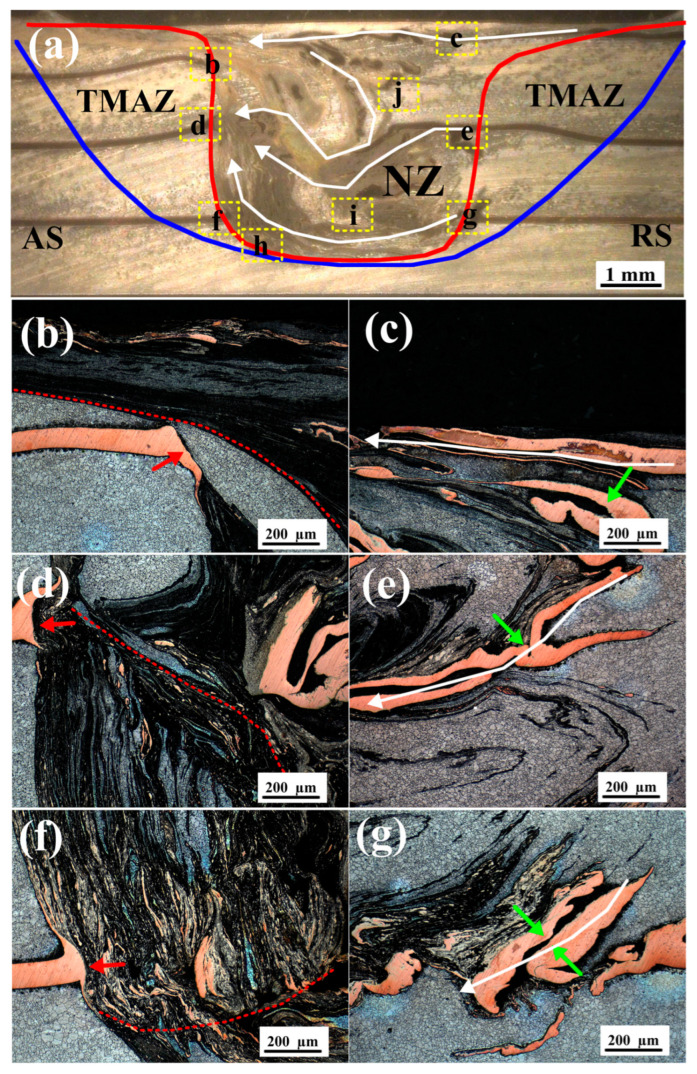
The macroscopic and local micrographs of the specimen C1 at the cross-section in the middle stage of the welded joint, which generally reveals the details of material flow in the vertical direction: (**a**) macroscopic optical image at the cross-section, and (**b**–**g**) local micrographs of the selected characteristic positions in different zones of the cross-section weld. In Figure 4a, the area within the red line represents the NZ, and the region between the blue line and the red line denotes the TMAZ.

Studying the distribution of plasticized metallic materials at the cross-section of the weld during the stable stage under FSW process is helpful to understand the materials flow behavior in the vertical direction in each affected zone of the welded joint, thereby enabling an effective judgment of defect locations. Figure 4 shows the macroscopic and local micrographs of the specimen C1 at the cross-section in the middle of the welded joint, which generally reveals the details of material flow in the vertical direction. It can be clearly seen that a typical transverse cross-section of the weld is given in Figure 4a, featuring the nugget zone (NZ) and the thermal–mechanical affected zone (TMAZ). Due to the presence of the marker material, i.e., copper foil, the material flow in NZ displays a complex characteristic pattern, in where the matrix material flow is affected by different parts of the stirring tool, roughly corresponding to the positions of the three layers of copper foils. The most distinct flow pattern is that the material surrounding the stirring tool is pulled from the retreating side (RS) to the advancing side (AS) of the weld and deposited at the rear of the tool in the NZ (indicated by the white solid lines with arrows in Figure 4a). It is worth noting that the first layer of copper foil basically flows from the RS to the AS in a simple “vector” manner, while the flow patterns of the other two layers of copper foils are more complex, involving a significantly vertical mixing effect. Eventually, the two parts of the material tend to converge at the middle layer of copper foil on the AS (as marked by the circular dashed box in Figure 4a), where defects may occur, such as tunnel, vacancy and so on. This result is consistent with numerous previous reports [10,35]. Hence, it can be considered that during the FSW process, the material flow throughout the weld appears a distinct directivity: the material in front of the rotating tool is transported to the rear and deposited from the RS to the AS. In order to clarify the flow behavior of the plasticized materials, it is necessary to conduct further analysis on the characteristic points in the NZ.

Figure 4b–g are the optical micrographs of each layer of copper foils at the cross-section of the C1 weld, with their specific positions indicated by the corresponding yellow dashed boxes in Figure 4a. In general, the fracture surfaces of copper foils on the AS of the weld are relatively smooth (as indicated by the red arrows in Figure 4b,d,f), featuring a downward-bending characteristic. With the increase in depth from the upper surface, the deformation length of all copper foils decreases gradually. It is thus easy to understand that the material on the AS undergoes intense shear deformation under the action of the rotating stirring tool. The decrease in the deformation length of the copper foils indicates that the interaction effect of the rotating shoulder is stronger than that of the stirring pin. Similar conclusions have been reported in many studies [8,36]. Additionally, it is noteworthy that near each copper foil on the AS of the weld, the plasticized metal presents a distinct boundary, and the material flow patterns above and below the boundary exhibit obvious differences (as shown by the red dashed lines in Figure 4b,d,f). Generally speaking, a typical friction stir weld cross-section consists of different zones: the shoulder-affected zone, the nugget zone with a ring/layered structure, and the swirl zone. These different zones are the major features typically found in welds with a distinct boundary between each other [16,37]. Although some suggestions on the mechanisms have been given in [35,38], the formation mechanism of the nugget zone and the ring-layered structure has not been fully elucidated, which is also one of the main objectives in this study. Interestingly, an upward-bending characteristic can be clearly observed at the fractures of the second and third layers of copper foils (as indicated by red arrows in Figure 4d,f), where the material flow implies the existence of different behaviors. In contrast, the copper foils on the RS of the weld show no obvious fracture; instead, they exhibit the characteristic of an upward extrusion of the plasticized metal (as indicated by green arrows in Figure 4c,e,g). Furthermore, it can also be found that the copper foils are significantly elongated on the RS, extending toward the weld or the AS along a specific flow direction identified by the white solid lines in Figure 4c,e,g. These flow patterns that appeared by the marker material clearly indicate that during the FSW process, the material on the RS is mainly extruded by the plastically deformed material on the AS and drawn into the weld by the rotating tool. Details of material flow in the weld will be further analyzed and discussed in subsequent sections.

**Figure 5:**
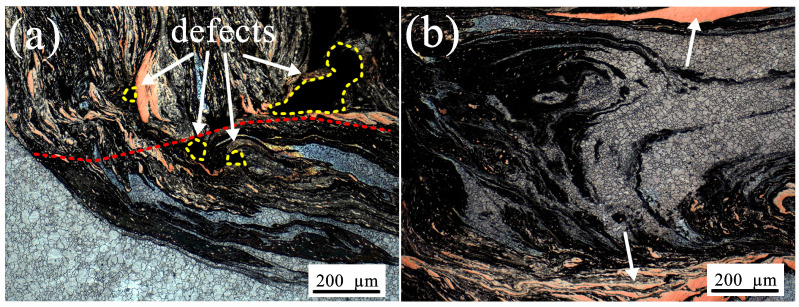
Optical images of marker material flow at specific positions inside the vertical weld, corresponding to points h and i in Figure 4: (**a**) the material flow and the defects at point h and (**b**) the “onion ring”-like structure at point i.

It is necessary to pay special attention to several areas, one of which is position h illustrated in Figure 4a, corresponding to the micrograph shown in Figure 5a. As shown in Figure 5a, several small void defects (one of which is larger than the others) can be observed on the bottom cross-section of the weld specimen, and these defects are located near the boundary marked by the red dashed line. As already mentioned above, distinct material flow behaviors give rise to the formation of different zones (here regarded as the nugget zone and swirl zone), leading to well-defined boundaries and indicating the presence of stagnant flow in this region. The elimination of these void defects once again requires a significantly higher level of plastic flows, namely, enhancing the material flow from the RS to the AS at the bottom of the weld, thereby enabling more material to fill this region [31]. The presence of these void defects provides a good indication of material flow directions in general.

In addition, another interesting point is position i shown in Figure 4a, which corresponds to the micrograph presented in Figure 5b. As shown in Figure 5b, an “onion ring”-like structure is formed in this region, located between the upper and lower layers of relatively intact copper foils (as indicated by the white arrows). By comparison, the position of the lower copper foil corresponds to that of the bottom copper foil on the RS, and the flow pattern from the RS to the AS can be clearly observed. Interestingly, no obvious large copper particles or copper foil strips are observed within the onion-ring structure; instead, only extremely fine copper particles are embedded in the dark-colored matrix. As analyzed above, the copper foils on the AS are fragmented into fine particles under shear action and drawn into the weld, whereas the copper foils on the RS undergo extrusion and stacking. This asymmetric mechanical behavior between the AS and RS has been clearly reported in Refs. [2,3,14]. Under such intense shear deformation, large copper particles are unlikely to exist in the AS material that enters the weld, which is instead dominated by a mixture of fine copper particles and Mg. This is consistent with the observation in Figure 5b. In addition, Chen et al. [7] demonstrated that the layer thickness of the “onion ring” is approximately equal to the ratio of welding speed to rotational speed (v/ω), which agrees with the shear-dominated mechanical characteristics of the AS material. As a consequence, it can be inferred that the lamellar geometry of the “onion ring”-like structure is mainly dominated by the material on the AS, while the material on the RS only plays an extrusion role. Although the formation mechanism of the “onion ring” structures has not been fully elucidated in the existing literature, several hypotheses regarding its underlying mechanism have been proposed in relevant studies, among which the shear lamination hypothesis is the most representative [16].

#### 3.2.2. Longitudinal Flow

**Figure 6:**
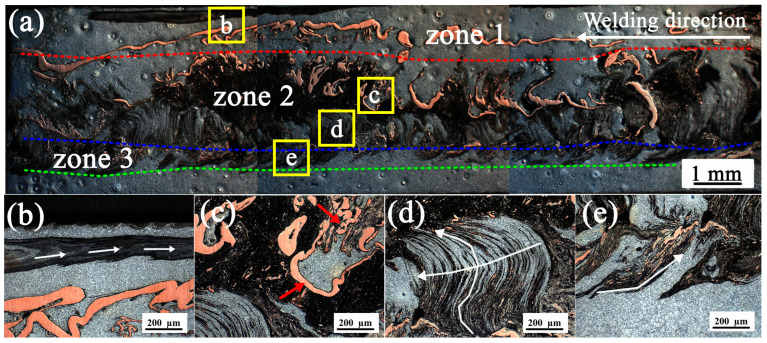
The general and local optical micrographs of the longitudinal cross-section, which is much closed to the AS along the weld center in the middle of the whole welded joint during the stable stage: (**a**) flow patterns along the longitudinal direction, and (**b**–**e**) the selected characteristic positions in different zones within the longitudinal weld.

Figure 6 presents the general and local optical micrographs of the longitudinal cross-section, which is much closer to the AS along the weld center in the middle of the whole welded joint (stable stage). As shown in Figure 6a, by identifying the marker material (copper foil) and the flow characteristics of the plasticized metal in the weld center, it can be found that the longitudinal cross-section of the weld can be clearly divided into three zones from top to bottom in the vertical direction: the horizontal laminar flow zone on the upper surface (zone 1), the complex flow zone in the middle (zone 2) that is attributed to the influence of the rotating shoulder and the stirring pin together, and the swirl flow zone at the bottom of the stirring pin (zone 3). These three zones also indirectly verify the aforementioned conclusion that the material flow along the vertical direction of the weld stagnates near the boundaries.

Specifically, the deformed copper foils in zone 1 appear an almost continuous horizontal strip-like morphology, indicating that the flow of plasticized metal here is a type of simple horizontal laminar flow. Figure 6b is the metallographic micrograph obtained by observing the characteristic position b selected in zone 1 (as shown in Figure 6a) for further observation. It is found that the plasticized metal material in the mixed gray region also shows a horizontal flow pattern (as indicated by the white arrows in Figure 6b). This phenomenon is fairly straightforward to interpret: as the stirring tool rotates, the friction between the tool shoulder and the metal on the upper surface of the workpiece generates heat, which softens the metal and causes it to adhere to the relatively rigid shoulder. A portion of the softened metal is driven by the rotating shoulder to undergo simple axial movement and is deposited in the cavity at the rear of the tool, thus yielding the characteristic of simple horizontal laminar flow on the longitudinal cross-section of the weld [18].

Figure 6c,d show the optical micrographs corresponding to positions c and d selected in zone 2 (Figure 6a). As mentioned above, the material flow in zone 2 is rather complex, which is also evidenced by the widely distributed marker material (i.e., copper foil). As shown in Figure 6c, the deformation of the marker material is highly irregular, indicating that the material in this zone undergoes an intensive plastic deformation process. A close examination of zone 2 in Figure 6a reveals that the deformation characteristics of the copper foils in this zone exhibit an “upward- or downward”-bending deformation, ultimately forming the characteristic geometric “C”-shaped traces as indicated by the red arrows in Figure 6c. The deformation characteristics demonstrate that the plasticized metal in this zone tends to shift obliquely along the welding direction. It is generally accepted that the material in the region encompassing position c is subjected to the combined effects of the shoulder and the pin. When the plasticized metal interacts with the shoulder and the pin, it deflects around the RS of the pin. Meanwhile, the material in front of the pin is extruded upward in the upper region of the workpiece, forcing the material to flow into the cavity beneath the tool shoulder. In the lower region, a small amount of material flows downward in front of the pin. These vertical movements occur simultaneously to the tangential flow around the RS of the pin [39], thereby forming the geometric “C”-shaped trace of the copper foil in the longitudinal directions. Moreover, Figure 6c shows how the continuous copper foil strips are disrupted as they flow within the weld, consequently fracturing into small, curled segments. This phenomenon may be attributed to the extensive deformation occurring along the rotating path, coupled with the insufficient elongation of the copper foil, which leads to fracture, curling, and accumulation.

Interestingly, although located in the same zone (zone 2), the material flow at position d presents a completely different flow pattern, as shown in Figure 6d. Distinct vertical material flow characteristics can be identified in this zone, where the plasticized metal is deposited layer by layer within the weld (as indicated by the white lines in Figure 6d). In this region, the interaction between the stirring pin and the material is quite stable, while the influence of the tool shoulder on the material becomes negligible. Some material undergoes shear deformation and rotates alongside the pin, forming the so-called “shear layer” [16,39]. Within this layer, the deformed material moves vertically under the action of the pin threads and then detaches as a single layer along the trailing edge during each rotation cycle. This detachment appeared to be quite complete (Figure 6d), which may imply that little material remains in the shear layer to continue rotating with the pin. During subsequent tool rotations, the detached layers are continuously deposited along the welding direction while the tool advances forward (with more layers deposited sequentially), thus forming the flow pattern observed in Figure 6d. It is worth noting that the “onion ring”-like structure mentioned above also appears in this zone (Figure 5b), indicating that the formation of the “onion ring”-like structure is strongly related to the stable and orderly deposition of the shear layer at the rear of the tool. As is well known, FSW is a thermo-mechanically coupled process, and temperature exerts a significant influence on material flow and weld formation [3,14]. At the initial stage, due to low heat generation and insufficient softening of the material, the formation of a stable material flow is unrealistic under the FSW process. As a result, it is difficult to form a stable and distinct “onion ring” structure during this stage [40]. In fact, the varying influence of the tool shoulder on material flow at different depths of the weld gives rise to the occurrence of different flow patterns within the same zone (Zone 2), which will be elaborated in Section 3.4.

Figure 6e is the optical micrograph of positions e selected in zone 3 (Figure 6a). In zone 3 near the root of the pin, relatively intact copper foil strips are barely detectable, which is replaced by an extensive shearing and thinning of the copper foil presenting in the layer structure. This indicates that the material in zone 3 is filled via layered deposition similar to the mechanism described above, leading to a relatively uniform dispersion of the copper foil within the shear layers or the material flow driven by the shear layers. At the lower levels close to the pin root, the flow is affected by both the roundness of the tool tip and the root gap, which enforce extrusion underneath the pin, as well as a constraint against material flow because of the back plate, therefore resulting in the production of the flow pattern of the plasticized metal, as shown in Figure 6e.

### 3.3. Horizontal Flow—Configuration C2

**Figure 7:**
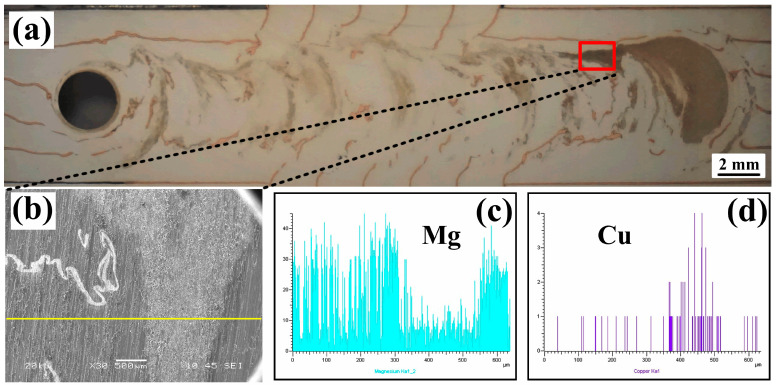
Energy dispersive spectrometer (EDS) analysis results of different flow regions on the horizontal welded plane at a distance of d = 1.0 mm below the upper surface. (**a**) The horizontal welded plane; (**b**) EDS scanned region denoted by the yellow line; (**c**,**d**) the elemental distribution results of Mg and Cu.

To better observe the flow behavior of the marker material along the horizontal direction, the polished specimens were exposed to air for several days. Oxidation occurred on the specimen surface due to contact with oxygen, which led to more distinct color differences between different regions. As shown in Figure 7a, this is one of the optical macrographs obtained at a location d = 1.0 mm slightly below the upper surface. It can be seen from Figure 7a that there is a distinct distribution of deformed copper foils and gray regions in the weld. A specific region on the weld (marked by the red box in Figure 7a) was selected for linear scanning using an energy dispersive spectrometer (EDS) to clarify the elemental distribution in the gray regions. The scanning results are shown in Figure 7c,d. From the line scan analysis, it can be observed that the intensity of Mg is much higher outside the gray region, but decreases sharply inside the gray region (Figure 7c), which means the lower percentage of Mg contained in the gray region. In contrast, the intensity of Cu increases significantly in the gray region (Figure 7d). Thus, it can be inferred that the gray regions in Figure 7a are a mixture of Mg and Cu. Therefore, the distribution of the gray regions in the horizontal weld corresponds to the distribution of the mechanical mixture composed of the marker material (copper foil) and AZ31 Mg alloy.

**Figure 8:**
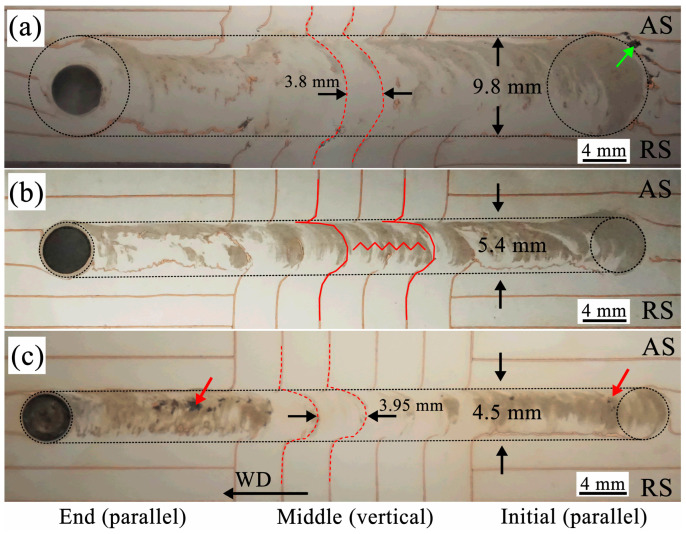
The final overall flow views of three horizontal sections of the FSW weld located at different depths from the top surface (d = 1.0, 2.0, and 3.0 mm) under the C2 condition: (**a**) d = 1.0 mm, (**b**) d = 2.0 mm, and (**c**) d = 3.0 mm.

Figure 8 shows the final flow views of three horizontal sections of the FSW weld located at different depths (d = 1.0, 2.0 and 3.0 mm) from the top surface under the C2 condition. Since two embedded directions (parallel and vertical) of copper foils in the whole weld are conducted, three research areas are artificially selected in the weld, which are named for the initial (parallel), the middle (vertical), and the end (parallel), as shown in Figure 8. It can be seen in Figure 8 that the width values of the welded joint formed by the traverse movement of the tool (shoulder and pin) gradually decrease from top to bottom, which are 9.8 mm (d = 1.0 mm), 5.4 mm (d = 2.0 mm), and 4.5 mm (d = 3.0 mm). Especially at the depth d = 2 mm, the width is significantly reduced, with a reduction of 81% compared to the depth d = 1 mm. Hence, the influence of the shoulder on the material flow is limited to the top surface of the weld, where the shoulder flow is the main flow feature. At d = 2.0 mm, the influence of the shoulder is already weak, where the weld width is almost equal to the diameter of the pin. This zone is often referred to as the stirring pin influence zone, where the material flow is mainly driven by the pin. At the location close to the bottom of the weld, the width of the weld is reduced slightly. Due to the existence of the tilted angle, the material flow here is affected by both the pin shape and the root gap [41]. Therefore, the material flow throughout the weld is driven by different parts of the rotating tool, which resulted in the formation of the obvious differences of the material flow patterns by marker materials in the horizontal plane at different depths, as shown in Figure 8. Based on the above, several particularly interesting phenomena can be seen, which will be discussed in detail later.

First, the marker materials running through the entire weld are mainly distributed from the middle to the advancing side, while, close to the retreating side, no or very little marker materials are observed, which is more obvious in the shoulder influence area (Figure 8a). Closer to the bottom of the weld, there are more marker materials on the retreating side. The distribution of the marker materials in the weld further indicates that the tool shoulder influence area is significantly restricted to the top surface of the workpiece (d = 1.0 mm), where it promotes the material transfer of the AZ31 magnesium alloy from the retreating to the advancing side in the weld [18]. This transfer of material at the top surface of the weld is clearly visible in Figure 4a and Figure 8a, where the marker materials can be seen at the top of the weld, in both the transverse and horizontal sections. It is worth noting that the marker materials are significantly distributed on both sides at the beginning of the FSW initial stage (indicated by the black dotted circles at the initial stage in Figure 8). This is most likely due to the fact that the tool is being continuously rotated in place for 20 s after inserting the workpiece according to the given program. During the period, the rotating shoulder constantly drives the material under it to move like the rigid shear flow, resulting in the production and accumulation of a large amount of Mg-Cu mixture. At the advancing side of the horizontal weld, some discontinuous welding defects can be clearly seen (indicated by the arrows in Figure 8a,c). Although the regions are different, they are all related to the termination of material flow on the AS (mentioned in Section 3.2.1). To eliminate these defects, the plastic flow of the material can be improved by optimizing welding parameters and other means [42].

In addition, during the intermediate stage of welding, it can be observed how the materials in different affected zones separate along the specific trailing edge and deposit in the weld at the rear of the tool after each rotation of the tool. In the affected zones of the shoulder and root, the final flow paths of the marker material (copper foils) in the weld are indicated by the red dashed lines in Figure 8a,c. Among them, the distances of the separation layers deposited by two adjacent copper foils are approximately 3.8 mm (Figure 8a) and 3.95 mm (Figure 8c), which are basically consistent with the initial embedding distance (4.0 mm). This indicates that the relative deposition position of the material in front of the tool remains basically unchanged after entering the weld, and the material undergoes significant deformation under shearing action. It is worth noting that the deposition trajectory on the individual copper foil is very complete and distinct, and there is little to no marker material between two adjacent separation layers. This likely implies that nearly the marker material was deposited almost simultaneously after rotating multiple turns with the tool. However, in the pin-affected zone, the deposition trajectory between adjacent copper foils is almost continuously distributed (indicated by the red wavy lines in Figure 8b), which apparently means that most material in the pin-affected zone rotates less than one full circle with the tool. It can be considered that the mode of separation and deposition is quite uniform, occurring almost in each rotation [36].

**Figure 9:**
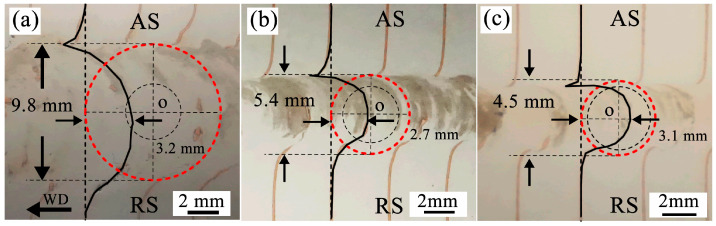
Magnified macrographs of the weld with marker material placed vertically along the transverse welding direction, showing the flow behavior of marker materials at different depths from the upper surface (d = 1.0, 2.0, and 3.0 mm): (**a**) d = 1.0 mm; (**b**) d = 2.0 mm; and (**c**) d = 3.0 mm. The red dashed circle indicates the weld zone, the black curve represents the flow trajectory of the marker material, and the black dashed circle denotes the stirring pin.

In more detail, Figure 9 shows the magnified macrographs of the final marker material flow in the weld at different depths (d = 1.0, 2.0, and 3.0 mm) in the middle stage of Figure 8. Here, the copper foils are vertically embedded along the welding travel direction, which can characterize the extension of the material deformation inside the weld, that is, the boundary between the highly deformed position close to the tool and the slightly deformed position far from the tool [39]. It can be considered that the highly deformed position is that where the marker material first contacts with the tool, while the slightly deformed position is that where the marker material deposits at the tail of the weld. The relative distance between these two positions can provide the deformed pattern and flow path that occurs after the material dragged into the weld. As can be seen in Figure 9a, within the shoulder-affected zone (d = 1 mm), the relative distance between the highly deformed position and the slightly deformed position is approximately 3.2 mm, which is about 33% of the whole weld width (9.8 mm, indicated by the red dotted circle), and the trace of the marker material is shown in Figure 8a. In contrast, in the pin-affected zone (d = 2 mm, as shown in Figure 9b) and the weld root zone (d = 3 mm, as shown in Figure 9c), the relative distances between these two positions are 2.7 mm and 3.1 mm, respectively, accounting for approximately 50% and 69% of the whole weld width (5.4 mm and 4.5 mm). This difference suggests the actual flow path that the material is driven to drag into the weld cavity and fully fills it by using the rotating tool at different depths. In the shoulder-affected zone (d = 1 mm), the softened advancing-side material is driven by the shoulder into the rotational zone at a position still far from the stirring pin and is deposited at the rear of the tool after rotating along an approximately elliptical trajectory (Figure 9a, denoted by the black curve). It can be seen that the deposition of the forward marker material is completed before it aligns with the center of the welding tool. In contrast, within the pin-affected zone (d = 2 mm), the effect of the shoulder is significantly weakened. Driven by the shearing action of the stirring pin, the marker material flows into the weld and completes deposition, with its trajectory forming a circle with a radius of 2.7 mm, as shown in Figure 9b (denoted by the black curve). Given that the weld width here is approximately 5.4 mm, it can be concluded that the marker material is nearly aligned with the center of the welding tool when the deposition is completed. In addition, the trace of the marker material between adjacent copper foils in this zone exhibit a relatively continuous distribution. This indicates that during the process of entering the weld, the material in the pin-affected zone first flows into the outermost transition zone, then moves into the rotational zone and enters the shear layer, and finally returns to the transition zone where it is ultimately deposited [3]. Finally, in the region close to the weld root zone (d = 3 mm), the deposition trajectory of the marker material is shown in Figure 9c (denoted by the black curve). It can be observed that the deposition is still incomplete when the marker material aligns with the center of the welding tool. Here, the material flow is subject to the dual effects of pin-tip roundness and root gap, both of which enhance the extrusion beneath the pin and impose constraints on the material flow due to the presence of the base plate [43]. Notably, the trajectory of the copper foils at the same position remains almost unchanged in the weld. This indicates that the material flow rate is not consistent along the depth direction of the entire weld; the closer the material is to the shoulder, the higher its flow rate.

**Figure 10:**
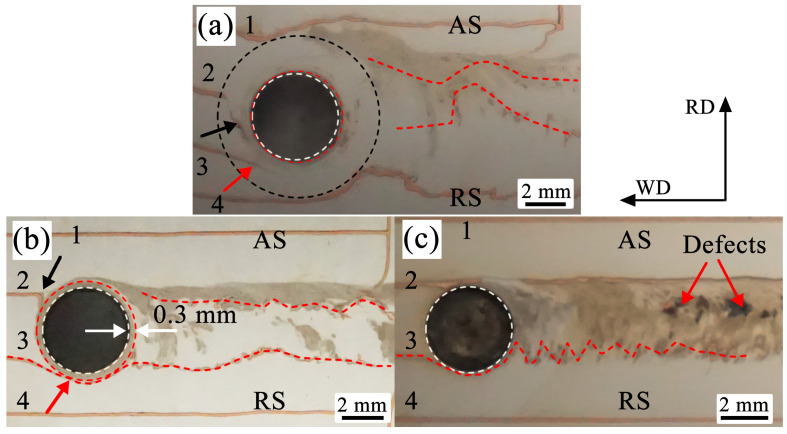
The magnified macrographs of the final flow behavior of the marker material near the pin hole in the weld at different depths from the upper surface (d = 1.0, 2.0, and 3.0 mm): (**a**) d = 1.0 mm; (**b**) d = 2.0 mm; and (**c**) d = 3.0 mm. The numbers represent the numbering of the copper foil from the AS to RS, the red dashed curve indicates the flow trajectory of the marker material, and the white dashed circle denotes the stirring pin.

Figure 10 presents the magnified macrographs of the final flow behavior of the marker material near the pin hole in the weld at different depths (d= 1.0, 2.0, and 3.0 mm) in the terminal stage of Figure 8. In this experiment, the copper foils were embedded parallel to the welding travel direction. As can be seen in Figure 10, only around the pin hole in the pin-affected zone (d = 2 mm) exists a distinct gray layer encapsulated by the marker material (as indicated by the red dashed circle in Figure 10b), with a thickness of approximately 0.3 mm. In contrast, such a layer is barely observable around the pin hole in both the shoulder-affected zone (d = 1 mm, as shown in Figure 10a) and the weld root zone (d = 3 mm, as shown in Figure 10c), especially in the region ahead of the welding tool. Based on the above analysis, this gray layer is a product of the thorough mixing between the copper foil and the Mg matrix, indicating that the material undergoes severe plastic deformation under the shearing action of the stirring pin, and a stable rotational flow zone is formed adjacent to the interface between the pin and the matrix. According to the reports of previous researchers, this stable flow zone is referred to as the shear layer, which consists of the transition zone and the rotational zone [3,16]. Based on the rotation speed (1500 rpm) and welding speed (50 mm/min) in this work, it can be roughly calculated that in such a shear layer with a thickness of 0.3 mm, the material on the advancing side in front of the tool rotates approximately 9 cycles with the tool from being dragged into the weld to being finally deposited in the cavity at the weld trailing edge. This case is impossible, as it is highly unlikely for the material to rotate multiple revolutions in the pin-affected zone (Figure 8b). Therefore, it is only possible that the material exchange on the AS occurs at the edge of the shear layer, while the material flow near the pin surface remains in a stable state [10]. It should be noted that since the “emergency stop” technique was not employed, the tool inevitably underwent repeated rotations, which resulted in a slight increase in the thickness of the shear layer around the pin hole. However, this does not preclude the inference drawn from the above result.

In addition, Figure 10 also illustrates how the material in front of the tool flows into the weld at different depths. In the shoulder-affected zone (d = 1 mm, as shown in Figure 10a), the copper foil on the AS in front of the pin hole (marked as No. 2) bends along the rotation direction of the tool (as indicated by the black arrow in Figure 10a). It even directly bends from the AS to the RS. Under the strong shear action of the shoulder, the copper foil eventually fractures into small, curved segments that enter the weld. A portion of these segments’ mixes with the Mg matrix, while another portion deposits in the weld in the form of small pieces. On the RS, the copper foil (marked as No. 3) is extruded by the material ahead of the tool transported from the AS to the RS and then dragged into the weld at rear of the tool (as indicated by the red arrows in Figure 10a). Actually, in the shoulder-affected zone (d = 1.0 mm), the shoulder-driven flow dominates the material flow behavior. The softened material ahead of the weld is dragged into the weld by the shoulder at a certain distance from the stir pin and then performs a circumferential motion at the interface between the shoulder and the matrix. In addition, in the pin-affected zone (d = 2.0 mm, as shown in Figure 10b), the copper foil on the AS (marked as No. 2) in front of the pin hole undergoes an obvious deflection of nearly 90° under the rotation of the stirring pin (as indicated by the black arrow in Figure 10b). It directly moves from its original position into the shear layer, where the mixing and exchange of the material take place. Meanwhile, the copper foil on the RS deflects around the pin, along the pin rotation direction, depositing on the RS at the trailing edge of the tool (as indicated by the red arrow in Figure 10b). Dominated by extrusion, the copper foil is deposited almost completely in the wake of the weld, shown in the red curve in Figure 10b. However, in the weld root zone (d = 3.0 mm, as shown in Figure 10c), the marker material is widely distributed throughout the weld, which serves as evidence of the complex deposition behavior. Here, the flow is jointly governed by both the shape of the pin root and the backing plate, which enhance downward extrusion, thereby imposing a constraint against the material flow, easily inducing welding defects at the weld root (as indicated by the red arrows in Figure 9c) [41]. It should be pointed out that the marker material initially positioned along the welding travel direction eventually forms similar S- or C-shaped paths after depositing material into the weld (including the copper foils splitting into small convoluted segments under shear action), as shown by the red dashed curves in Figure 10. It can be predicted that, if material flow occurs only in two-dimensional directions, i.e., the flow induced by the rotating cylindrical surface has a non-zero velocity component in only one direction, it would be a stable symmetric flow, then the material would be deposited at the same horizontal position as it entered. In fact, this is rather difficult because of the influences by the shoulder, threads, pin root, or backing plate, leading to the asymmetry of the flow [8].

Furthermore, it can be seen that continuous copper foil is split into small, convoluted segments on the RS, but do not appear on the AS. This indicates that a “shear asymmetry” phenomenon exists in the shear deformation zone in the FSW process, where shear deformation is much stronger on the AS than that on the RS, as previous reports by some researches show [2,38].

### 3.4. Flow and Force

#### 3.4.1. Definition of Characteristic Forces

The stress analysis of the material in weld during FSW is extremely complex, as it involves the coupling of multiple physical fields such as high temperature, large deformation, material flow, and phase transformation. In addition, the stress state and flow pattern of the material are mutually determined and inseparably coupled. Force is the driving factor of flow, while flow serves as a direct manifestation of force distribution. Based on the experimental studies proposed in this work, and drawing on theories and research on material mechanics and fluid mechanics [31,44], a “force-flow coupling” framework is introduced to understand the complex flow phenomena within the FSW weld. Here, to simplify the complexity of the problem, the following basic assumptions are made:

1. The materials is treated as a rigid viscoelastic body (neglecting elastic deformation and focusing on plastic flow);
2. It is assumed that the material flow within the weld is in a steady state and behaves as a non-Newtonian incompressible fluid (relative to the moving heat source);
3. The material flow behavior is described using fundamental constitutive equations from material mechanics and fluid mechanics.

**Figure 11:**
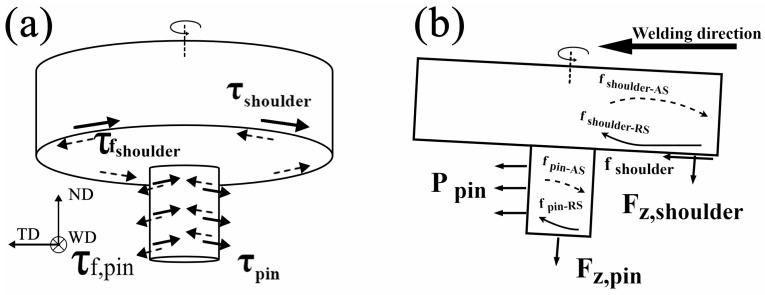
Schematic diagram of force analysis at the interface between the material and the rotating tool during FSW process: (**a**) force analysis normal to the welding direction, and (**b**) lateral force analysis.

The internal stress field (force) within the material directly drives and shapes its flow patterns. Therefore, the three-dimensional force distribution experienced by the material during the FSW process is presented firstly in this work, as illustrated in Figure 11, which is defined as follows:

- τ_shoulder_: The circumferential shear stress generated by the rotation of the shoulder is one of the primary driving forces for material flow in the weld;
- τ_pin_: The circumferential shear stress generated by the rotation of the stirring pin drives the material to undergo shear flow;
- τ_f, shoulder_: The viscous friction force generated between the flowing material and the shoulder surface that acts in the opposite direction to the shear force τ_shoulder_;
- τ_f, pin_: The viscous friction force generated between the flowing material and the pin surface that acts in the opposite direction to the shear force τ_pin_;
- F_z, shoulder_: The downward force applied by the shoulder along the spindle direction;
- F_z, pin_: The downward force applied by the root of the pin along the spindle direction;
- P_pin_: The forward pressure exerted by the stirring pin on the unwelded material ahead along the welding direction, which can also be regarded as the advancing resistance F_x_, acting in the opposite direction.

In fact, the stress state of the material is more complex than illustrated in Figure 11 during the entire welding process. Factors such as the pin geometry, shoulder surface characteristics and workpiece conditions can impact the material flow. For general analysis and understanding, the above simplifications are made to facilitate the study.

#### 3.4.2. Establishment of Mathematical Expression

When the tool performs a rotational circular motion, this process can be regarded as the torsional deformation of a circular shaft. When a circular shaft is subjected to an external torsional couple acting around its axis, the shaft will undergo torsional deformation. Its cross-section remains planar and experiences only rigid rotation [45]. Hence, according to the compatibility equation of deformation, the following expression is obtained:

(1) $$\tau \left(\rho \right)=32\frac{M_{\omega }}{\pi d^{4}}\rho$$

This equation represents the expression for the shear stress on the cross-section of a circular shaft with diameter *d*. Here, *τ* denotes the shear stress, *ρ* is the distance from any arbitrary point on the cross-section to its center, *M_ω_* is the rotational torque, *ω* is the angular velocity of the shaft rotation, and *d* is the diameter of the circular shaft.

**Figure 12:**
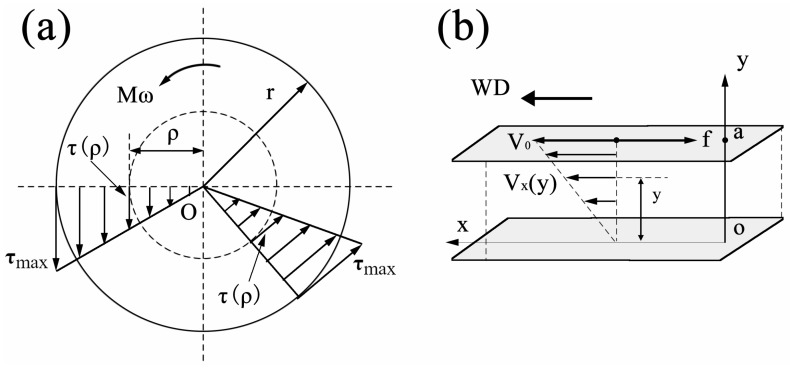
Illustration of (**a**) shear stress distribution on the cross-section under circumferential motion and (**b**) velocity distribution under simple shear flow.

Equation (1) indicates that, for a circular shaft with a diameter of d and a rotational angular velocity of *ω*, the shear stress at each point on its cross-section is proportional to the distance from that point to the center of the cross-section. In other words, the shear stress exhibits a linear distribution along the radius of the cross-section, with its direction as illustrated in Figure 12a.

Integrating Equation (1) over the cross-section with an area of *A*, the torque acting on this cross-section can be obtained:

(2) $$\int_{A}\rho \tau \left(\rho \right)dA=M_{x}$$

In contrast, when the shoulder and the pin perform rotational circular motion, the shear stress acting on the plasticized material will generate an oppositely directed frictional shear stress *τf* at the tool–workpiece interface. Considering that during the steady welding stage, a stable viscoelastic boundary layer (shear layer) is formed between the pin/shoulder and the workpiece, the “solid friction” at the tool–workpiece interface can be simplified as the “shear transfer” between adjacent viscous fluids [31]. Therefore, when the tool travels at a constant welding speed *V*, the material flow between the shoulder and the bottom of the workpiece can be simplified as simple shear flow. It should be pointed out that this is an idealized condition.

As shown in Figure 12b, the fluid flow is induced by the movement of the upper plate (the shoulder). Under steady-state conditions (i.e., after a sufficiently long time has elapsed since the upper plate began moving), the velocity of the fluid increases linearly from 0 at the lower plate (the bottom of the workpiece) to *V*_0_ at the upper plate (the shoulder), which can be described by the following equation:

(3) $$v_{x}\left(y\right)=V_{0}\frac{y}{a}$$

This type of flow is generally referred to as simple shear flow or plane Couette flow. In the formula, *V*_0_ is the initial constant velocity of the upper plate; *a* is the distance between the two plates. It can be concluded that in the aforementioned shear flow, an analogous relationship exists between the frictional force f and the velocity gradient between the two plates (*f* is directed along the negative direction of the x-axis):

(4) $$\frac{f_{x}}{S}=\mu \frac{V_{0}}{a}=-\mu \frac{dv_{x}}{dy}$$

where $f_{x}$/*S* is referred to as shear stress (i.e., frictional shear stress *τf*); *S* is the effective area of the frictional force acting on the plate; and *μ* is the dynamic viscosity coefficient of the fluid, or viscosity for short, which can be obtained by the following equation [44].

(5) $$\mu \approx \frac{h}{\alpha }e^{\frac{∆g_{0}}{k_{B}T}}$$

where *k_B_* is the Boltzmann constant, *T* is the temperature, Δ*g*_0_ is the activation energy, *h* is the Planck constant, and *α* is a coefficient with the dimension of volume. Equation (5) shows that the viscosity of the liquid decreases with increasing temperature.

Equation (4) characterizes the distribution of shear stress in a one-dimensional direction. Without considering convection for the time being, this equation can be extended to two-dimensional and three-dimensional cases, i.e., the above relationship remains valid under rotational conditions, with only the direction opposite to the rotational tangential direction. It can be inferred that the closer the position is to the shoulder, the larger the velocity gradient of the fluid and, consequently, the greater the shear stress.

#### 3.4.3. Calculation of Stress and Torque

**Figure 13:**
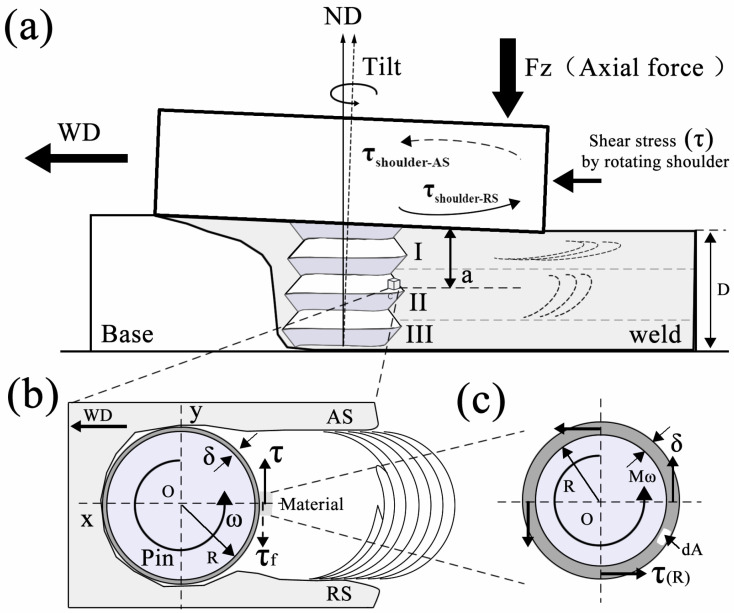
Illustration of (**a**) selected material on the surface of the pin under the FSW process; (**b**) stress analysis of the material flow in the x-y plane during FSW and (**c**) shear motion of materials in a two-dimensional film layer.

A two-dimensional cross-section is selected on the side surface of the pin at any depth within the weld to analyze the stress distribution of the material before it is deposited into the weld. Under the conditions of rotational speed *ω* and welding speed *V*, the following assumptions are proposed: in the steady welding stage, the material flow between the shoulder and the base plate follows simple shear flow, and a stable viscous fluid film with a thickness *δ* is formed on the pin side surface, as illustrated in Figure 13. At this point, the linear velocity on the pin surface is $V_{pin}=\omega R$, and the velocity component of the shear motion by the shoulder at this location is $V_{s}=\omega R\left(L-a\right)/L$ (approximately linear). The velocity of the material at the outer edge of the film is approximately zero (constrained by the surrounding rigid material). Here, *R* is the radius of the stirring pin; *L* is the effective length of the pin. Therefore, based on Equations (3) and (4), the shear stress is derived as:

(6) $$\tau =\mu \omega R\left(\frac{L-a}{L^{2}}+\frac{1}{\delta }\right)$$

This equation indicates that the shear stress on the side surface of the pin consists of two components, representing the effects of the shoulder and the pin, respectively. At this point, the magnitude of the shear force per unit area acting on the pin surface is equal to *τ*, and its direction is opposite to the rotational direction.

Substituting Equation (6) into Equation (2) and performing the integration, the total torque on the side surface of the pin can be obtained as follows:

(7) $$M_{pin}=\int_{A}R\tau dA\approx 2\pi RL\cdot \tau \cdot R=2\mu \pi R^{3}L\cdot \omega \left(\frac{L-a}{L^{2}}+\frac{1}{\delta }\right)$$

This simplified equation clearly shows that the pin torque *M_pin_* is proportional to the viscosity *μ* and the rotational speed *ω*, and inversely proportional to the thickness δ of the viscous layer. It is particularly worth pointing out that the term (*L* − *a*)/*L*^2^ in the parentheses demonstrates that the closer the position is to the shoulder, the larger the pin torque *M_pin_* will be. Since the viscosity *μ* itself is a function of strain rate $\dot{\gamma }$ and temperature *T*, Equation (7) reflects strong nonlinearity, representing a simplified “two-dimensional shear flow model”.

#### 3.4.4. Establishment of a Simple “Flow Around a Cylinder” Model

In this subsection, we aim to derive the basic flow state and distribution of the two-dimensional flow field under the condition of flow around a cylinder based on the fundamental theories of fluid mechanics.

If the velocity field of an ideal incompressible fluid flow varies only in two coordinate directions (two-dimensional flow), the following relationship exists between the velocity components of the two-dimensional flow and the stream function *Ψ*:

(8) $$v_{r}=\frac{1}{r}\cdot \frac{\partial \Psi }{\partial \varphi }=\frac{\partial \Phi }{\partial r}$$

(9) $$v_{\varphi }=-\frac{\partial \Psi }{\partial r}$$

**Figure 14:**
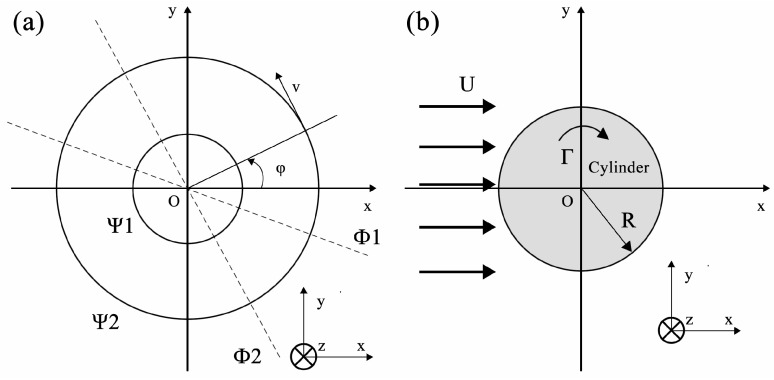
Schematic diagram of plane point vortex streamlines around the z-axis and flow around a cylinder with circulation: (**a**) plane point vortex streamlines (*Ψ*1, *Ψ*2) and equipotential lines (Φ1, Φ2), (**b**) flow around a cylinder with circulation.

Here, the material flow can be characterized as a two-dimensional planer vortex flow, which rotates about an axis that is perpendicular to the x-y plane and passes through the origin *O*, as schematically shown in Figure 14a. At this point, the velocity components $v_{r}$ and $v_{\varphi }$ satisfy the following equations, respectively, in the polar coordinate system: $v_{r}=0$, $v_{\varphi }=\frac{\Gamma }{2\pi r}$, where Γ is a constant. Using Equations (8) and (9), we obtain:

(10) $$\frac{1}{r}\frac{\partial \Phi }{\partial \varphi }=-\frac{\partial \Psi }{\partial r}=v_{\varphi }=\frac{\Gamma }{2\pi r}$$

(11) $$\frac{\partial \Phi }{\partial r}=\frac{1}{r}\;\frac{\partial \Psi }{\partial \varphi }=v_{r}=0$$

where, *r* represents the radial vector; φ generally denotes the azimuthal angle in two-dimensional cases (i.e., the angle deviating from the positive direction of the x-axis in the x-y plane); and Φ stands for the velocity potential.

By calculating the velocity circulation along the circle (C) centered at *O* with radius *r*, the following result is obtained:

(12) $$\int_{∁}v\cdot dl=\int_{0}^{2\pi }\frac{\Gamma }{2\pi r}rd\varphi =\Gamma$$

Therefore, Γ is the circulation along any arbitrary curve enclosing the origin. The existence of this circulation causes the fluid to rotate around the center, *O*. Furthermore, it follows that:

(13) $$\Phi =\frac{\Gamma \varphi }{2\pi }$$

Next, based on the vortex flow, we analyzed the circulation-free flow around a cylinder, i.e., the flow induced by the disturbance of a uniform flow with velocity *U* by a cylinder of radius *R*, where the cylinder axis is perpendicular to the flow direction, as illustrated in Figure 14b. In this case, the velocity potential Φ of the flow field can be expressed as:

(14) $$\Phi =Ur\cos\varphi -\frac{p\cos\varphi }{2\pi r}=\left(Ur-\frac{p}{2\pi r}\right)\cos\varphi$$

where *p* denotes the dipole moment.

Finally, the velocity potential for the flow around a cylinder with circulation can be obtained by superimposing the potential function of a point vortex flow with circulation Γ (Equation (13)) onto Equation (14). For a uniform flow with velocity magnitude *U* parallel to the x-axis, the potential function of the flow around a cylinder with circulation is given by:

(15) $$\Phi =\left(Ur-\frac{p}{2\pi r}\right)\cos\varphi +\frac{\Gamma \varphi }{2\pi }$$

Hence, we obtain:

(16) $$v_{r}=U\left(1-\frac{R^{2}}{r^{2}}\right)\cos\varphi$$

(17) $$v_{\varphi }=-U\left(1+\frac{R^{2}}{r^{2}}\right)\sin\varphi +\frac{\Gamma }{2\pi r}$$

The above is the velocity distribution equation for the flow around a cylinder with circulation, which is also distribution equation for streamlines in the flow field. By analyzing this equation, a series of streamlines can be obtained. The following equation can be used to determine the shapes of a series of streamlines under different conditions and to identify whether stagnation points on the cylinder surface (points on the circumference of the cylinder where the flow velocity is zero) exist:

(18) $$v_{\varphi }\left(r=R\right)=-U\left(1+\frac{R^{2}}{R^{2}}\right)\sin\varphi +\frac{\Gamma }{2\pi R}=0$$

Which is:

(19) $$\sin\varphi =\frac{\Gamma }{4\pi RU}$$

**Figure 15:**
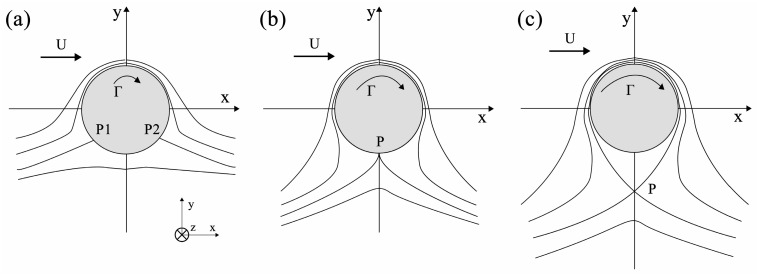
Schematic diagram of the streamline patterns in the flow field of the flow around a cylinder with circulation and the forces acting on the cylinder. It is assumed that the flow velocity is uniform at infinity, and the circulation is defined as negative in the figure: (**a**) 0<|Γ|<4πR|U|; (**b**) |Γ|=4πR|U|; and (**c**) |Γ|>4πR|U|.

According to the different values of the circulation magnitude |Γ| and the velocity magnitude |U| in Equation (19), two flow fields with distinct patterns can be obtained:

1. If 0<|Γ|<4πR|U|, two stagnation points, P1 and P2, exist on the cylinder surface, which are symmetric to the y-axis, as shown in Figure 15a Their positions are determined by the angle φ, which is the solution to Equation (19). As the circulation magnitude |Γ| increases, P1 and P2 gradually approach each other from diametrically opposite positions (in the case of circulation-free flow around the cylinder, |Γ|=0 and φ=0), and eventually merge into a single stagnation point P when |Γ|=4πR|U|, as illustrated in Figure 15b.
2. If |Γ|>4πR|U|, there are no stagnation points on the cylinder surface. However, closed streamlines can be observed in the flow field immediately adjacent to the cylinder surface, while the streamlines far away from the wall are open curves (Figure 15c). Additionally, a stagnation point P exists in the flow field outside the cylinder, whose coordinate φ satisfies sinφ=±1 (the sign depends on whether Γ and *U* have the same sign).

#### 3.4.5. Establishment of the Relationship Between Force and Flow

For the ideal flow around a cylinder with circulation, there exist two distinct flow regimes in the flow field, which are related to the different values of the circulation magnitude |Γ| and the velocity magnitude |U|. Herein, we combine the flow around a cylinder with circulation model with the two-dimensional simple shear flow model to qualitatively illustrate the influence of the relevant mechanical parameters in FSW on material flow behavior.

In cylindrical Couette flow model, the following relationship exists between the circulation Γ and the rotational speed *ω*:

(20) $$\Gamma =2\pi \omega \cdot R^{2}$$

Additionally, assuming the material flow during the stable friction stir welding stage is treated as a simple shear flow mode with a pressure gradient, the relationship between the average velocity *U* and the welding speed *V* can be simply expressed by the following equation:

(21) $$U=\frac{V}{2}-\frac{∆p}{D}\frac{a^{2}}{12\mu }$$

where △*p*/*D* is the pressure gradient of the flowing material in the x-direction, which can be interpreted as the advancing resistance *F_x_* per unit area in the x-direction; *D* is the diameter of the stirring pin, i.e., 2*R*. In Equation (21), *U* refers to the average flow rate per unit cross-sectional area and is opposite to *V* in direction. Thus, Equation (21) can be simplified as:

(22) $$U=-\frac{V}{2}+\frac{aF_{x}}{24\mu \pi R^{2}}$$

Substituting Equations (20) and (22) into Equation (19), we obtain:

(23) $$\sin\varphi =\frac{\Gamma }{4\pi RU}=\frac{12\mu \pi R^{3}\omega }{{aF}_{x}-12\mu \pi R^{2}V}$$

where Γ is defined as negative for a clockwise direction and positive for a counterclockwise direction.

This equation is referred to as the force-flow coupled simple flow model around a rotating cylinder. It exhibits obvious nonlinearity and directly demonstrates the effects of the advancing resistance *F_x_*, rotational speed *ω*, and welding speed *V* on material flow during the FSW process:

1. When $\left|\sin\varphi \right|=\left|\frac{12\mu \pi R^{3}\omega }{{aF}_{x}-12\mu \pi R^{2}V}\right|=1$, it follows that $\left|V\pm R\omega \right|=\left|\frac{aF_{x}}{12\mu \pi R^{2}}\right|$.

According to the flow around a cylinder with circulation mode, there exists a stagnation point P on the stirring pin, as shown in Figure 15b. At this point, the material dragging into the weld fills the cavity exactly before reaching the stagnation point P, resulting in a defect-free weld. This represents an ideal FSW state, where the material flow around the stirring pin is the superposition of uniform flow and vortex flow. Any variation in the rotational speed *ω* or welding speed *V* at this moment will break the equilibrium, causing the material flow to deviate from this state and trend to distribute in Mode A or Mode B.

2. When $\left|\sin\varphi \right|=\left|\frac{12\mu \pi R^{3}\omega }{{aF}_{x}-12\mu \pi R^{2}V}\right|<1$, if it rotates clockwise (*ω* is negative), then $\left|V-R\omega \right|>\left|\frac{aF_{x}}{12\mu \pi R^{2}}\right|$.

In this case, an increase in welding speed *V* or a decrease in rotational speed *ω* will cause the material flow to tend toward Mode A distribution. At this point, two stagnation points exist around the stirring pin, where the material flow velocity is zero. This results in insufficient material flow, which fails to fully fill the weld and thus leads to welding defects. Based on the streamline patterns in the flow field depicted in Figure 15a, it can be determined that the defects are mainly distributed on the advancing side of the weld. The higher the welding speed *V* or the lower the rotational speed *ω*, the closer the defects are to the center of the weld. It is also consistent with the actual conditions of defect formation during FSW process.

Conversely, when the rotational speed *ω* is increased or the welding speed *V* is decreased, the material flow tends toward the Mode B distribution. According to the flow field analysis results, there are no stagnation points around the stirring pin under this condition, and the material adjacent to the pin may undergo multiple revolutions. However, since |V−Rω| also has a nonlinear relationship with *Fx* and *μ*, for instance, an excessively high *ω* will lead to a significant decrease in *μ* [15], which limits the occurrence of such flow state. Eventually, the material flow in the stirring pin zone will return to a state close to the ideal FSW condition.

3. If it rotates counterclockwise (*ω* is positive), i.e., $\left|V+R\omega \right|>\left|\frac{aF_{x}}{12\mu \pi R^{2}}\right|$, the material flow will have a Mode B distribution, which cannot be sustained, for the reasons stated in (2).
4. In addition, when the rotational speed *ω* and welding speed *V* are fixed, substituting *a* = 0 into Equation (23) yields $\sin\varphi =-\frac{R\omega }{V}$

It can be seen from the above equation that *ω*/*V* has a maximum value at this position, which is ${\frac{\omega }{V}}_{max}=-\frac{\sin\varphi }{R}$. The material flow herein tends toward a Mode B distribution, meaning that more material undergoes multiple revolutions with the stirring pin, indicating better fluidity. It can be inferred that the temperature is the highest and the material flow is optimal at the junction of the shoulder and the pin (*a* = 0). However, when *a* = *L* (where *L* is the effective length of the stirring pin), |sinφ| decreases, indicating that the material flow state at this location tends toward a Mode A distribution. The presence of stagnation points leads to insufficient material flow at the bottom of the weld, which is prone to the formation of root defects.

It should be pointed out that the above conclusions are derived under the condition of an ideal fluid. In practice, the material flow during FSW process does not fully follow the flow behavior of an ideal fluid. Therefore, the above models and conclusions are only for qualitative reference.

## 4. Conclusions

In this study, conventional metallographic sectioning was adopted as the primary observation method, and copper foil was used as the marker material. The three-dimensional material flow behavior during the friction stir welding of AZ31 magnesium alloy was characterized via three configurations of the marker material. Combining the principles of material mechanics and fluid mechanics, a force-flow coupled simple flow model around a rotating cylinder was proposed. The main conclusions were drawn as follows:

1. Three characteristic zones exist along the vertical direction of the weld under the friction stir welding process, which are, from top to bottom, the shoulder-affected zone, the pin-affected zone, and the swirl zone.
2. The material flow patterns vary in three characteristic zones: the shoulder-affected zone is dominated by laminar flow, the pin-affected zone features complex mixed flow, and the swirl zone is characterized by rotational flow.
3. During the friction stir welding process, the material on the advancing side (AS) is mainly subjected to the shearing action of the shoulder and pin, while the material on the retreating side (RS) is primarily subjected to extrusion and dragging forces from the material on the AS. Only the material at the AS enters the shear layer, whereas the material at the RS rarely does so.
4. The proposed force-flow coupled “simple flow model around a rotating cylinder” model defines three flow modes, which correspond to the different characteristic zones within the weld.

## Data Availability

The original contributions presented in this study are included in the article. Further inquiries can be directed to the corresponding authors.
